# Microvesicles from bone marrow-derived mesenchymal stem cells promote *Helicobacter pylori*-associated gastric cancer progression by transferring thrombospondin-2

**DOI:** 10.1186/s12964-023-01127-y

**Published:** 2023-10-05

**Authors:** Cuihua Qi, Huiying Shi, Mengke Fan, Weigang Chen, Hailing Yao, Chen Jiang, Lingjun Meng, Suya Pang, Rong Lin

**Affiliations:** 1 Department of Gastroenterology, Union Hospital, Tongji Medical College, Huazhong University of Science and Technology, Wuhan, 4300222 China; 2 Department of Gastroenterology, The First Affiliated Hospital of Shihezi University, Shihezi, 832002 China

**Keywords:** Bone marrow mesenchymal stem cells (BMSCs), Microvesicles (MVs), Thrombospondin 2 (THBS2), Gastric cancer (GC), *Helicobacter pylori* (*H pylori*)

## Abstract

**Background:**

Our previous study found that bone marrow-derived mesenchymal stem cells (BMSCs) promote *Helicobacter pylori (H pylori)*-associated gastric cancer (GC) progression by secreting thrombospondin-2 (THBS2). Extracellular vesicles (EVs) are important carriers for intercellular communication, and EVs secreted by BMSCs have been shown to be closely related to tumor development. The aim of this study was to investigate whether BMSC-derived microvesicles (MVs, a main type of EV) play a role in *H. pylori*-associated GC by transferring THBS2.

**Methods:**

BMSCs and THBS2-deficient BMSCs were treated with or without the supernatant of *H. pylori* for 12 h at a multiplicity of infection of 50, and their EVs were collected. Then, the effects of BMSC-derived MVs and THBS2-deficient BMSC-derived MVs on the GC cell line MGC-803 were assessed by in vitro proliferation, migration, and invasion assays. In addition, a subcutaneous xenograft tumor model, a nude mouse intraperitoneal metastasis model, and a tail vein injection metastasis model were constructed to evaluate the effects of BMSC-derived MVs and THBS2-deficient BMSC-derived MVs on GC development and metastasis in vivo.

**Results:**

BMSC-derived MVs could be readily internalized by MGC-803 cells. BMSC-derived MVs after *H. pylori* treatment significantly promoted their proliferation, migration and invasion in vitro (all *P* < 0.05) and promoted tumor development and metastasis in a subcutaneous xenograft tumor model, a nude mouse intraperitoneal metastasis model, and a tail vein injection metastasis model in vivo (all *P* < 0.05). The protein expression of THBS2 was significantly upregulated after *H. pylori* treatment in BMSC-derived MVs (*P* < 0.05). Depletion of the THBS2 gene reduces the tumor-promoting ability of BMSC-MVs in an *H. pylori* infection microenvironment both in vitro and in vivo.

**Conclusion:**

Overall, these findings indicate that MVs derived from BMSCs can promote *H. pylori*-associated GC development and metastasis by delivering the THBS2 protein.

Video Abstract

**Supplementary Information:**

The online version contains supplementary material available at 10.1186/s12964-023-01127-y.

## Background

Gastric cancer (GC) is the 6th most common cancer and the 4th leading cause of cancer-related deaths worldwide [1]. *Helicobacter pylori (H. pylori)* is the main cause of noncardia GC and infects over half of the world’s population [2]. Mesenchymal stem cells (MSCs) are multipotent cells that can be isolated from a wide variety of tissues [3]. MSCs were reported to home to sites of tumor microenvironments and participate in the formation of the tumor microenvironment [4]. Multiple lines of evidence suggest that MSCs can promote tumor development and metastasis by developing into cancer-associated fibroblasts (CAFs) [5]. In addition, increasing evidence indicates that MSCs play an important role in promoting gastric cancer proliferation, survival, invasion and metastasis [6, 7]. Our previous study found that bone marrow-derived mesenchymal stem cells (BMSCs) can differentiate into both gastric epithelial cells and CAFs to participate in the development of chronic *H. pylori*-associated GC [8].

Extracellular vesicles (EVs) carrying substantial populations of bioactive molecules, including proteins, lipids, and nucleic acids, are important carriers for intercellular communication and circulating biomarkers [9]. Studies have shown that EVs are heterogeneous multisignal messengers that play an essential role in both primary tumor growth and metastatic evolution [10]. In addition, EVs may be biomarkers and novel therapeutic targets for cancer progression and can be used for disease diagnosis and prognostic evaluation [11]. Microvesicles (MVs) represent one of the major classes of EVs and are formed by the outward budding of the plasma membrane and are an important mediator for information exchange between donor cells and recipient cells [12]. ​MVs regulate many important cellular processes, including facilitating cell invasion, cell growth, and evasion of the immune response and stimulating angiogenesis, drug resistance and many other processes [13]. Previous studies have shown that MVs, as an important mediator of intercellular communication between cancerous and stromal cells, are involved in tumor immunosuppression, tumor metastasis, tumor-interstitial interactions, tumor angiogenesis and other processes [14, 15].

MSC-derived EVs, which include exosomes and MVs, are involved in cell-to-cell communication, cell signaling, and altering cell or tissue metabolism [16]. Studies have demonstrated that BMSC-derived EVs can activate the Hedgehog signaling pathway [17] and enhance vascular endothelial growth factor expression in tumor cells to promote tumor progression [18]. Moreover, exosomes derived from human BMSCs can enhance the migration and invasion of GC cells via the induction of epithelial‑mesenchymal transition [19]. However, the effects of BMSC-derived MVs on the progression of chronic *H. pylori-*associated GC remain unclear.

Our previous study found that BMSCs promoted the progression of chronic *H. pylori*-associated GC by secreting thrombospondin-2 (THBS2) [8]. THBSs are secreted glycoproteins that function in the extracellular matrix (ECM) [20]. THBS2 belongs to the family of matricellular Ca^2+^-binding glycoproteins secreted by stromal fibroblasts, endothelial cells, and immune cells. This protein has functions in inflammation, inhibits angiogenesis, and mediates ECM assembly [21, 22]. Studies have reported high expression of THBS2 in gastric cancer tissues and worse prognosis in patients with higher THBS2 expression [23, 24]. THBS2 is highly expressed in the stroma of gastric cancer patients [25].

Therefore, the aim of this study was to investigate whether BMSC-derived MVs promote the progression of *H. pylori*-associated GC by transferring THBS2.

## Methods

### Extraction and isolation of microvesicles

The MVs were extracted and isolated from the supernatants, as described in a previous study [26]. First, fetal bovine serum (FBS) used for cell culture was ultracentrifuged at 100,000 × g for 12 h to remove the extracellular vesicles (EVs). Then, exponentially growing cells were cultured in EV-depleted medium for 48 h. After incubation, the supernatants were centrifuged for 10 min at 600 g to remove the cells and centrifuged for 30 min at 2,000 g to remove the debris. Finally, the supernatant was centrifuged for 75 min at 16,000 g to pellet the MVs. These pelleted MVs were washed with phosphate-buffered saline (PBS) and centrifuged at 20,000 × g for 75 min to remove the residual soluble factors.

### Transmission electron microscopy

The prepared MVs were pipetted onto Formvar carbon-coated copper grids. Then, the adsorbed MVs were negatively stained with 2% (w/v) phosphotungstic acid (PTA, pH 6.8) for two minutes. Next, the grids were washed with distilled water and then air dried. The MVs were observed using a transmission electron microscope (Wuhan Institute of Virology, 100 kV Hitachi H-7000FA).

### Nanoparticle tracking analysis

The size and concentration of MVs were analyzed using nanoparticle tracking analysis (NTA). NTA was carried out using the ZetaView Multiple Parameter Particle Tracking Analyzer (Particle Metrix). The diffusion constant was calculated from the direct observation of Brownian motion and transferred into a distribution diagram of particle size and concentration via the Einstein-Stokes relation between the diffusion constant and particle size.

### Tumorigenesis in nude mice

Male BALB/c nude mice (4–6 weeks old) were purchased from Beijing Huafukang Biotechnology Co., Ltd., and bred in an SPF laboratory. These mice were randomly grouped as follows (*n* = 4): 1) Control: Control mice injected with PBS; 2) MGC-803: Mice injected with 2 × 10^6^ MGC-803 cells; 3) MGC-803 + BMSC-MVs: Mice injected with 2 × 10^6^ MGC-803 cells with BMSC-MV intervention; 4) MGC-803 + Hp + BMSC-MVs: Mice injected with 2 × 10^6^ MGC-803 cells with Hp + BMSC-MV intervention. Cells in 0.2 ml of PBS were subcutaneously injected into the right armpit region of BALB/c nude mice. The tumor size was measured every three days using calipers after transplantation. The tumor volume was calculated using the formula (L × W^2^)/2, where L is the length and W is the width of the tumor [27]. These mice were sacrificed 22 days after injection, and the subcutaneous tumors were analyzed by histological staining.

### Animal model of tumor metastasis assay in vivo

MGC-803 cells (2 × 10^6^) were subcutaneously injected into the right armpit region of BALB/c nude mice. The metastasis of subcutaneous tumors in nude mice was evaluated after three months with 18F-fluorodeoxyglucose positron emission tomography/computed tomography (18F-FDG PET/CT). In addition, 2 × 10^6^ MGC-803 cells were intraperitoneally or intravenously injected into the tail veins of mice. The metastasis of abdominal tumors and the colonization of tumor cells in nude mice were detected with 18F-FDG PET/CT (TransPET®BioCaliburn® LH, RAYCAN Technology Co., Ltd., Suzhou, China) and CT after four weeks.

### Lentivirus construction and transfection

The lentivirus vector expressing thrombospondin-2 (THBS2)-specific short hairpin RNA (shRNA) was constructed by Gene Technologies, Inc. (Shanghai, China). The nucleotide sequence of the THBS2-specific shRNA was gcTGTAGGTTTCGACGAGTTT. For stable knockdown of thrombospondin- 2 (THBS2) in BMSCs, lentiviral vectors at an MOI of 100 were added at a cell density of 60%. The supernatant was discarded 24 h after infection and replaced with complete fresh medium. Stably transfected cell lines were selected using puromycin (Sigma‒Aldrich; Merck Millipore) at a dose of 8 μg/ml for 3 days. The efficiency of lentivirus interference was detected by PCR and western blotting.

### Analysis of the effect of THBS2-deficient BMSC-MVs on tumor growth and metastasis

For evaluation of the effect of THBS2-deficient BMSC-MVs on tumor growth and metastasis in the *H. pylori* infection microenvironment, BALB/c nude mice were allocated to the following groups: 1) Control: PBS transplantation group; 2) MGC-803: MGC-803 transplantation group; 3) MGC-803 + NC-BMSC-MVs: MGC-803 with negative control BMSC-MV (NC-BMSC-MV) intervention; 4) MGC-803 + Hp + NC-BMSC-MVs: MGC-803 with Hp + NC-BMSC-MV intervention; 5) MGC-803 + sh-THBS2-BMSC-MVs: with THBS2-specific shRNA BMSC (sh-THBS2-BMSC-MV) intervention; and 6) MGC-803 + Hp + sh-THBS2-BMSC-MVs: MGC-803 with Hp + sh-THBS2-BMSC-MV intervention.

### Statistical analysis

All experiments were conducted at least three times. Statistical analysis was performed using SPSS 21.0 software. All data are presented as the mean ± standard deviation (SD). The significant differences were analyzed by one-way ANOVA or independent-sample t test. *P* < 0.05 was considered statistically significant. In the present study, *P < 0.05, **P < 0.01, and ***P < 0.001.

Information on the protocols used for culture of *H. pylori*, characterization of BMSCs, micro-PET/CT imaging in vivo, HE staining and Giemsa staining are as described in our previous research [8]. The methods for cell culture, CFSE-labeling assay, transwell migration assays, qRT‒PCR, western blot analysis, immunohistochemistry and immunofluorescence are provided in the Supporting Information.

## Results

### Characterization of microvesicles

**Fig. 1:**
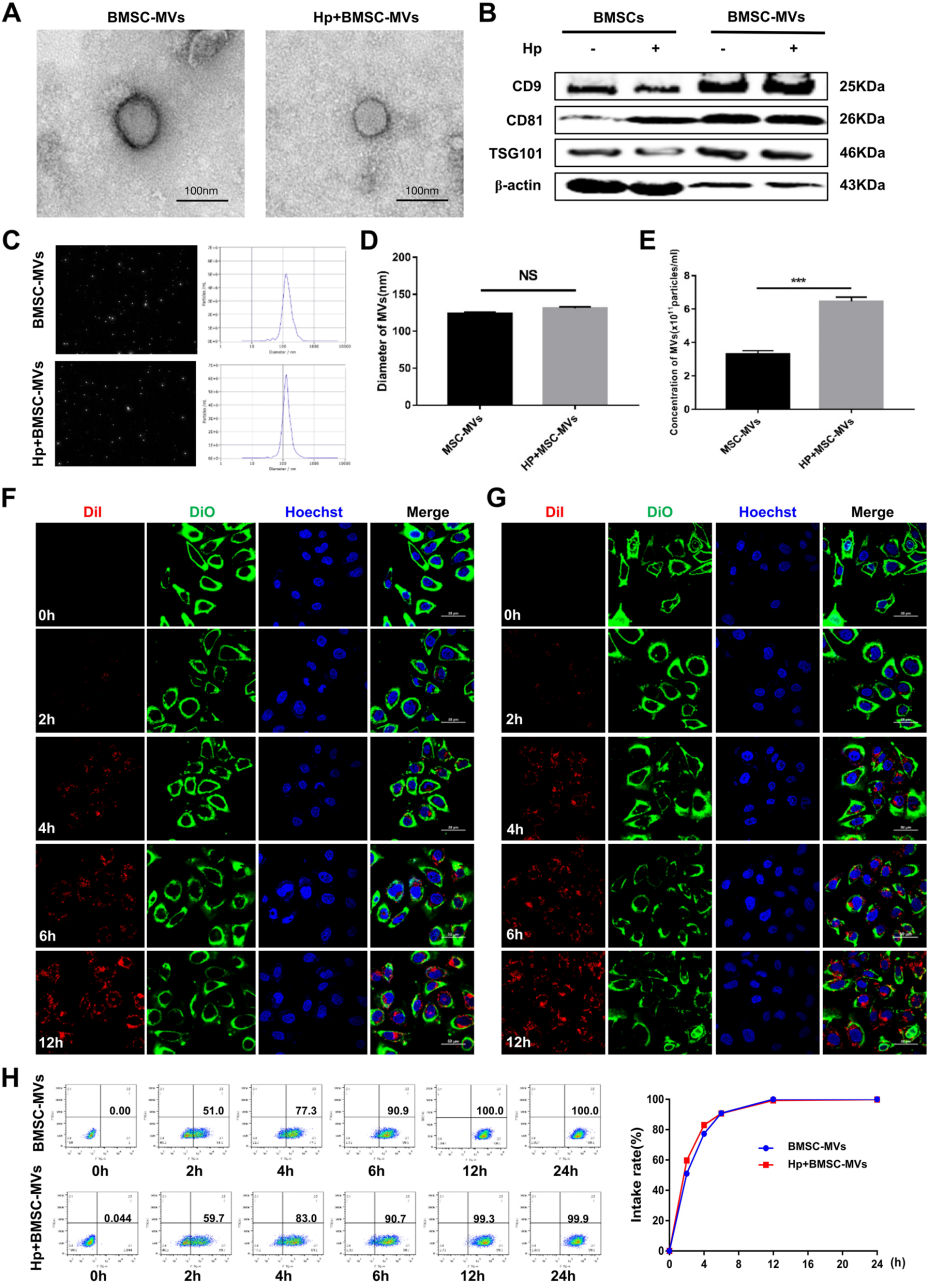
Characterization of microvesicles. **A** Transmission electron microscopy images of BMSC-MVs and Hp + BMSC-MVs; scale bar = 100 nm. **B** Representative immunoblot bands of CD9, CD81 and TSG101 in BMSCs and BMSC-MVs. **C**-**E** Diameter and concentration of microvesicles analyzed by NTA. **F** Representative immunofluorescence confocal laser images of BMSC-MVs (red) and MGC-803 (green), the nuclei (blue), Scale bar = 50 μm. **G** Representative immunofluorescence confocal laser images of Hp + BMSC-MVs (red) and MGC-803 (green), the nuclei (blue), scale bar = 50 μm. **H** The intake rate of BMSC-MVs and Hp + BMSC-MVs in MGC-803 cells analyzed by FCM. BMSCs: bone marrow-derived mesenchymal stem cells; MVs: Microvesicles; Hp: *Helicobacter pylori;* GC: gastric cancer; NTA: nanoparticle tracking analysis; FCM: flow cytometry

Ultracentrifugation was used to extract and separate BMSC-MVs and Hp + BMSC-MVs from BMSCs and BMSCs exposed to *H. pylori*, respectively. Transmission electron microscopy revealed that BMSC-MVs and Hp + BMSC-MVs were disc-shaped or goblet-shaped membranous vesicles with a complete cell membrane (Fig. 1A). Western blot analysis revealed that the protein expression of extracellular vesicle markers (CD9, CD81 and TSG101) in BMSC-MVs and Hp + BMSC-MVs was positive (Fig. 1B). Nanoparticle tracking analysis was performed to identify BMSC-MVs and Hp + BMSC-MVs, and the MVs exhibited a round or oval morphology with a diameter of 120–135 nm (Fig. 1C, D). Moreover, the concentration of MVs derived from Hp + BMSCs was significantly higher than that of BMSCs (*P* < 0.001, Fig. 1E).

### Uptake and internalization of MVs

To determine whether BMSC-MVs can transfer biological information to MGC-803 cells, we detected the uptake and internalization of MVs in MGC-803 cells with immunofluorescence and flow cytometry (FCM). Immunofluorescence results showed that the MVs (red) were mainly distributed in the cell membrane (green labeled) after 12 h of cocultivation (Fig. 1F-G). FCM results showed that there was no difference in the uptake rate of BMSC-MVs and Hp + BMSC-MVs in MGC-803 cells, and almost all MVs were taken up by gastric cancer cells after 12 h (Fig. 1H). Therefore, these findings indicated that MGC-803 can almost completely take up BMSC-MVs within 12 h.

### Microvesicles derived from BMSCs with *H. pylori* intervention enhance the proliferation, migration and invasion of MGC-803 cells in vitro

**Fig. 2:**
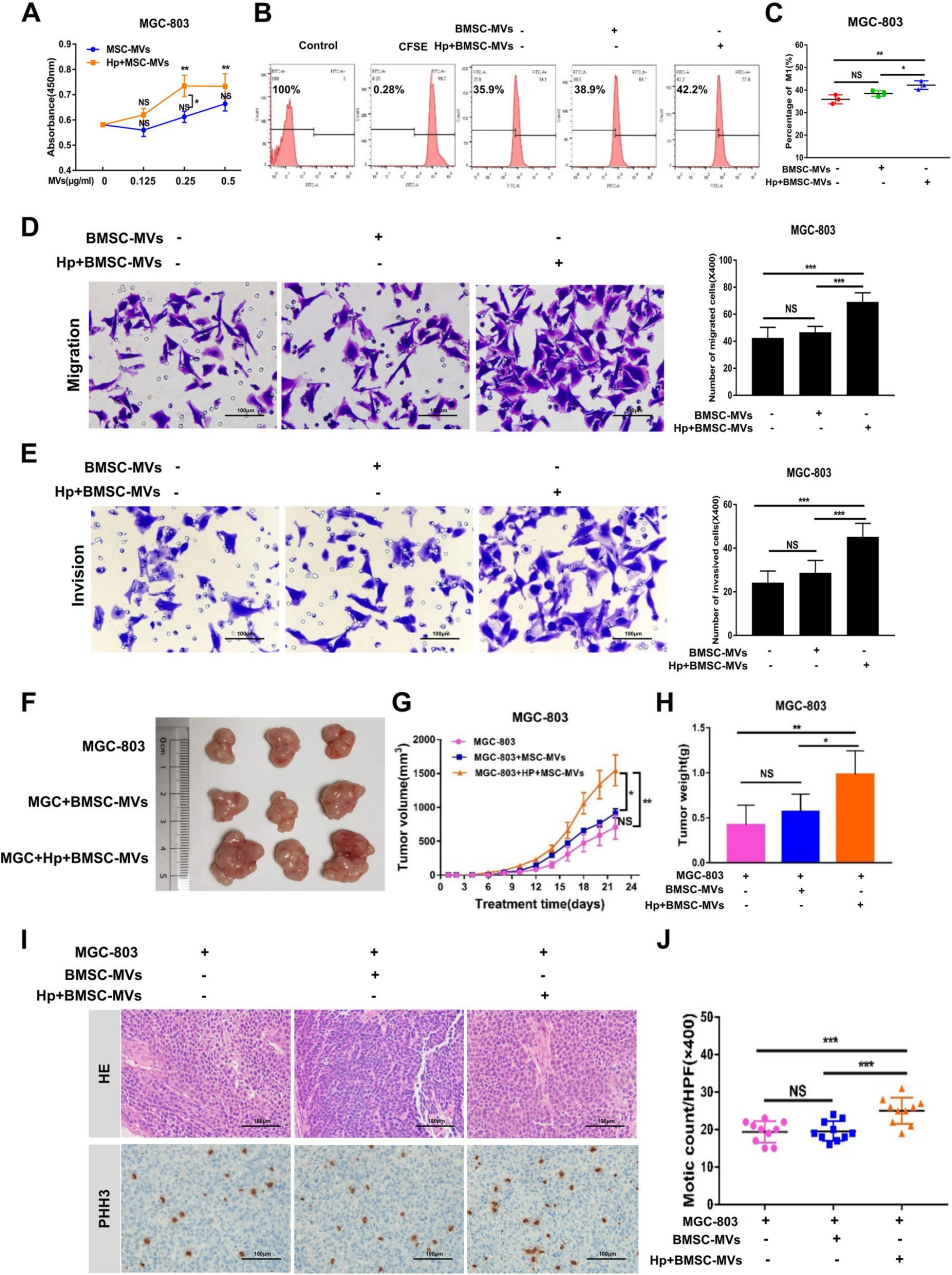
MVs derived from BMSCs with *H. pylori* intervention enhance the proliferation, migration and invasion of MGC-803 cells in vitro and promote the development of GC in nude mouse xenografts. **A**-**C** The proliferation of MGC-803 cells with or without BMSC-MV/Hp + BMSC-MV intervention. **D** The migration of MGC-803 cells with or without BMSC-MV/Hp + BMSC-MV intervention. **E** The invasion of MGC-803 cells with or without BMSC-MV/Hp + BMSC-MV intervention. **F**–**H**. Representative images of tumor, tumor size and tumor weight in nude mice 3 weeks after injection of MGC-803 cells alone, MGC-803 cells cocultured with BMSC-MVs, and MGC-803 cells cocultured with Hp + BMSC-MVs. **I** Representative PHH3 IHC images and quantitative analysis of the PHH3 index of tumor sections from MGC-803 cells alone, MGC-803 cells cocultured with BMSC-MVs, and MGC-803 cells cocultured with Hp + BMSC-MVs are shown. BMSCs: bone marrow-derived mesenchymal stem cells; MVs: microvesicles; Hp: *Helicobacter pylori;* GC: gastric cancer; IHC: immunohistochemistry. *P < .05, **P < .01, ***P < .001

To determine the role of BMSC-MVs in the proliferation of MGC-803 cells, we treated cells with different concentrations of BMSC-MVs (0, 0.125, 0.250 and 0.500 μg/ml) for 12 h. The cell counting kit-8 assay results indicated that the MVs derived from BMSCs exposed to *H. pylori* significantly enhanced the proliferation of MGC-803 cells at 0.25 μg/ml (Fig. 2A, *P* < 0.05). Similarly, the results of the CFSE assay showed that the proliferation rate of MGC-803 cells was significantly increased after Hp + BMSC-MV (0.25 μg/ml, 12 h) intervention (Fig. 2B, C, *P* < 0.01). Cell migration and invasion were further examined using the Transwell system. The results showed that Hp + BMSC-MVs significantly promoted the migration (Fig. 2D, *P* < 0.001) and invasion (Fig. 2E, *P* < 0.001) of MGC-803 cells in vitro. In addition, there was no significant difference between the BMSC-MV intervention group and the control group in the proliferation, migration and invasion of MGC-803 cells in vitro (Fig. 2B-E, *P* > 0.05).

### MVs derived from BMSCs with H. pylori intervention promote the development and metastasis of GC in nude mouse xenografts

The role of BMSC-MVs in the tumorigenesis of *H. pylori*-associated GC was evaluated using a subcutaneous xenograft tumor model in nude mice. Three weeks after injection, the weight and volume of the subcutaneous tumor in the Hp + BMSCw-MVs + MGC-803 group were significantly greater than those in the BMSC-MVs + MGC-803 (*P* < 0.01) and MGC-803 groups (*P* < 0.05, Fig. 2F-H). Immunohistochemical staining showed that the number of mitotic cells in the Hp + BMSC-MVs + MGC-803 group was significantly higher than that in the other groups (***P < 0.001, Fig. 2I, J).

**Fig. 3:**
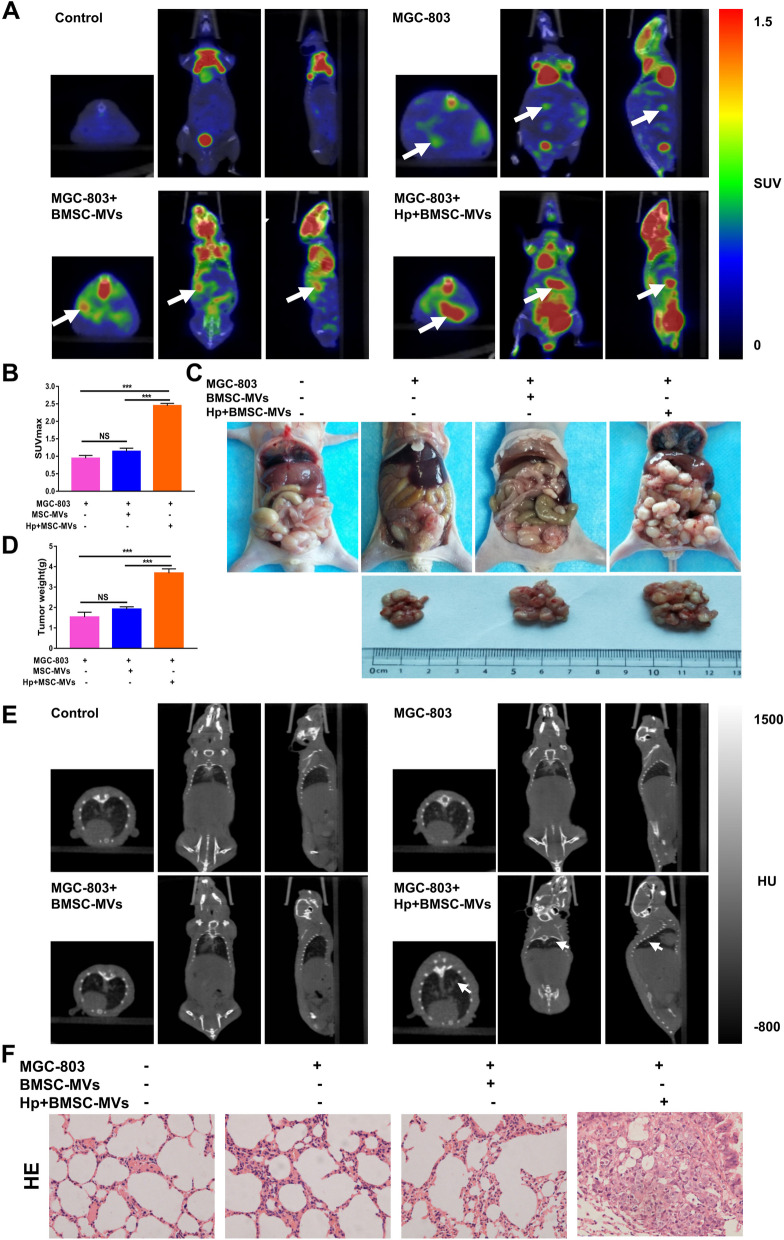
MVs derived from BMSCs with *H. pylori* intervention promote the metastasis of GC in nude mice xenografts in vivo. **A** Representative PET-CT images of nude mice 4 weeks after intraperitoneal injection with MGC-803 cells alone, MGC-803 cells cocultured with BMSC-MVs, and MGC-803 cells cocultured with Hp + BMSC-MVs. The white arrow indicates suspected abdominal metastasis of xenograft tumors. **B** The SUV_max_ values of intraperitoneal metastases in different groups of nude mice. **C**, **D** Representative images and tumor weights of intraperitoneal metastatic tumors in different groups of nude mice. **E** Representative CT images of nude mice 4 weeks after tail vein injection with MGC-803 cells alone, MGC-803 cells cocultured with BMSC-MVs, and MGC-803 cells cocultured with Hp + BMSC-MVs. **F** H&E pathological images of lung tissue 4 weeks after tail vein injection in different groups of nude mice. BMSCs: bone marrow-derived mesenchymal stem cells; MVs: microvesicles; Hp: *Helicobacter pylori;* GC: gastric cancer; ***P < .001, NS: no significance

To evaluate the effect of BMSC-MVs on the metastasis of *H. pylori*-associated GC, we generated a nude mouse abdominal cavity metastasis model. Four weeks after transplantation, abdominal metastasis was detected in the transplantation group using micro PET-CT, and glucose metabolism in the Hp + BMSC-MVs + MGC-803 group was higher than that in the other groups (*P* < 0.001, Fig. 3A, B). Moreover, the size and weight of irregular tumors in the abdominal cavity were higher in the Hp + BMSC-MV + MGC-803 group than in the other groups (*P* < 0.001, Fig. 3C, D). A tail vein injection metastasis model was constructed to evaluate the colonization of tumor cells in nude mice. Four weeks after transplantation, CT examination found suspicious lung nodules in the Hp + BMSC-MVs + MGC-803 group, and the pathological results were tumors (Fig. 3E, F). Taken together, these results suggested that BMSC-MVs can promote *H. pylori*-associated GC progression and metastasis.

### MVs derived from BMSCs exposed to *H. pylori* express higher protein levels of THBS2

**Fig. 4:**
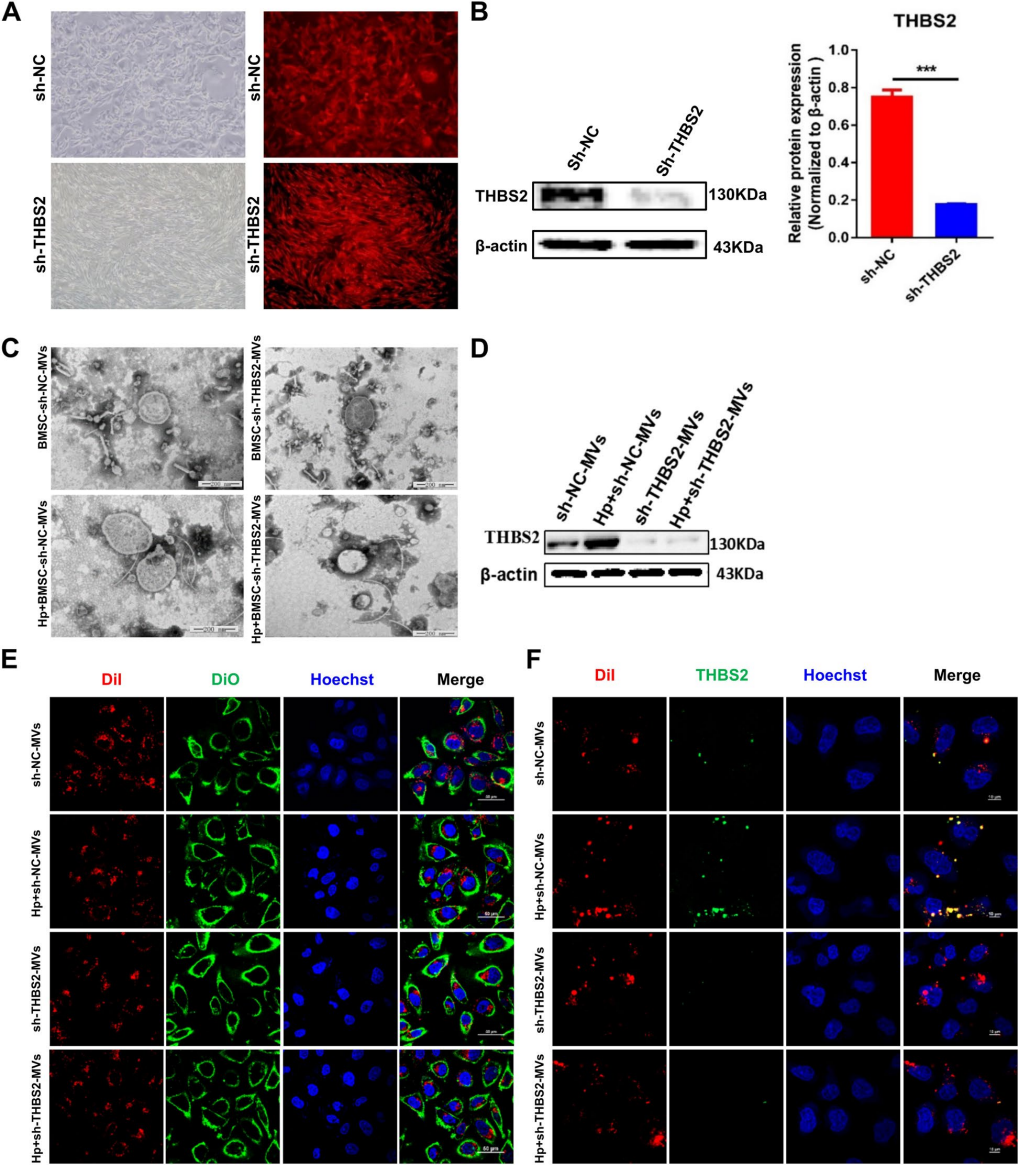
MVs derived from BMSCs exposed to *H. pylori* express higher protein levels of THBS2. **A** BMSCs were transfected with scrambled shRNA (negative control) or THBS2 shRNA. Transfection was confirmed by the presence of red fluorescent protein. **B** Western blotting confirmation of successful THBS2 depletion following THBS2-specific shRNA transfection (sh-THBS2) compared with NC transfection (sh-NC). **C** Transmission electron microscopy images of sh-NC-MVs, Hp + sh-NC-MVs, sh-THBS2-MVs and Hp + sh-THBS2-MVs; scale bar = 50 nm. **D** Representative immunoblot bands of THBS2 in sh-NC-MVs, Hp + sh-NC-MVs, sh-THBS2-MVs and Hp + sh-THBS2-MVs. **E** Representative immunofluorescence confocal laser images of BMSC-MVs (red) and MGC-803 cells (green) and the nuclei (blue), scale bar = 50 μm. **F** Representative immunofluorescence confocal laser images of BMSC-MVs (red) and THBS2 (green), the nuclei (blue), Scale bar = 50 μm. BMSCs: bone marrow-derived mesenchymal stem cells; MVs: microvesicles; Hp: *Helicobacter pylori;* GC: gastric cancer; NC: negative control. **P < .01, ***P < .001, ****P < .0001

Our previous research found that BMSCs can promote *H. pylori*-associated GC progression by secreting thrombospondin-2 (THBS2) [8]. We further investigated the expression level of THBS2 in BMSC-MVs and HP + BMSC-MVs. Western blot analysis and immunofluorescence analysis showed that the protein expression of THBS2 in BMSC-MVs was significantly upregulated after exposure to *H. pylori* (*P* < 0.5, Fig. 4D, 4F). Therefore, we transfected BMSCs with THBS2-specific shRNA, and the western blot results showed that THBS2 protein expression was significantly downregulated in Sh-THBS2-BMSCs and Sh-THBS2-BMSC-MVs compared with Sh-NC-BMSCs and Sh-NC-BMSC-MVs, respectively (*P* < 0.001, Fig. 4A, B, 4D). After *H. pylori* intervention, THBS2 protein expression was significantly upregulated in Sh-NC-BMSC-MVs (*P* < 0.001; Fig. 4D, 4F) but not in Sh-THBS2-BMSC-MVs (*P* > 0.05; Fig. 4D, 4F). The characterization of MVs was performed with transmission electron microscopy, nanoparticle tracking analysis, and western blotting (Fig. 4C, Supplementary Fig. 3). Laser confocal microscopy was used to observe the uptake and internalization of MVs by MGC-803 cells (Fig. 4E). MVs (red) specifically labeled with THBS2 (green) were co-cultured with MGC-803 for 12 h, and the result showed that MVs can transfer THBS2 to MGC-803 (Fig. 4F).

### Depletion of the THBS2 gene in BMSC-MVs inhibits the proliferation, migration and invasion of MGC-803 cells in vitro

**Fig. 5:**
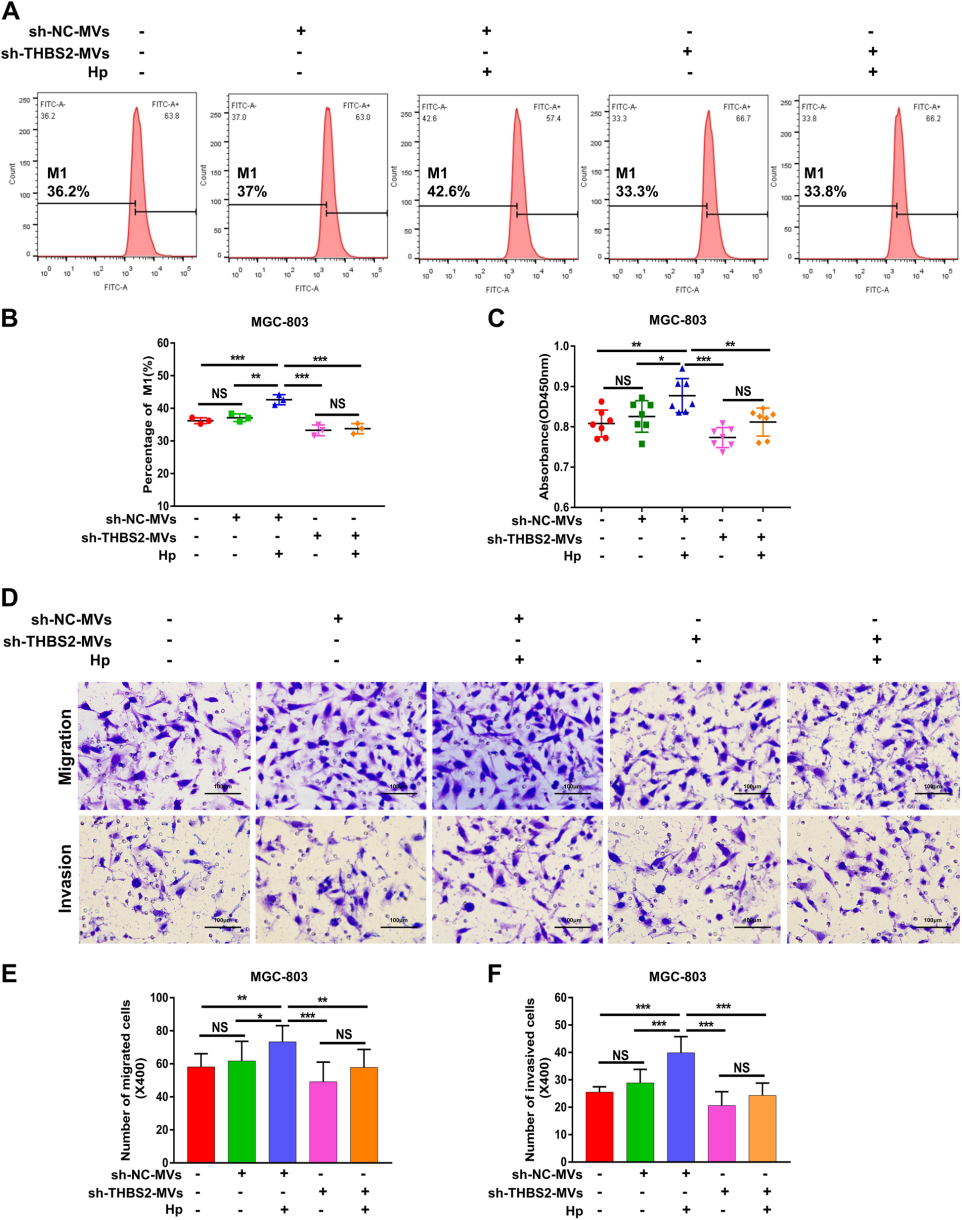
Depletion of the THBS2 gene in BMSC-MVs inhibits the proliferation, migration and invasion of MGC-803 cells in vitro. **A**-**C** The proliferation of MGC-803 cells cocultured with sh-NC-MVs/sh-THBS2-MVs with or without *H. pylori* intervention. **D**-**F** The migration and invasion of MGC-803 cells cocultured with sh-NC-MVs/sh-THBS2-MVs with or without *H. pylori* intervention. BMSCs: bone marrow-derived mesenchymal stem cells; MVs: microvesicles; Hp: *Helicobacter pylori;* GC: gastric cancer; IHC: immunohistochemistry. *P < .05, **P < .01, ***P < .001, NS: no significance

Next, the effects of sh-THBS2-BMSC-MVs on the proliferation, migration and invasion of MGC-803 cells were evaluated. The results of the CFSE assay showed that the proliferation of MGC-803 cells in the sh-THBS2-BMSC-MVs + HP group was significantly reduced compared with that in the sh-NC-BMSC-MVs + HP group (Fig. 5A, B, *P* < 0.001). The results of the CCK-8 assay for cell proliferation were consistent with those of the CFSE assay (Fig. 5C). Moreover, the Transwell assay revealed that the MVs derived from sh-THBS2-BMSCs + HP inhibited the migration (Fig. 5D, E, *P* < 0.01) and invasion (Fig. 5D, F, *P* < 0.001) of MGC-803 cells compared with the MVs derived from sh-NC-BMSCs + HP. These results indicated that HP + BMSC-MVs promote the proliferation, migration and invasion of MGC-803 cells in vitro by transferring THBS2, while knockdown of THBS2 in BMSCs can reduce this effect.

### Depletion of the THBS2 gene reduces the tumor-promoting ability of BMSC-MVs in nude mouse xenografts

**Fig. 6:**
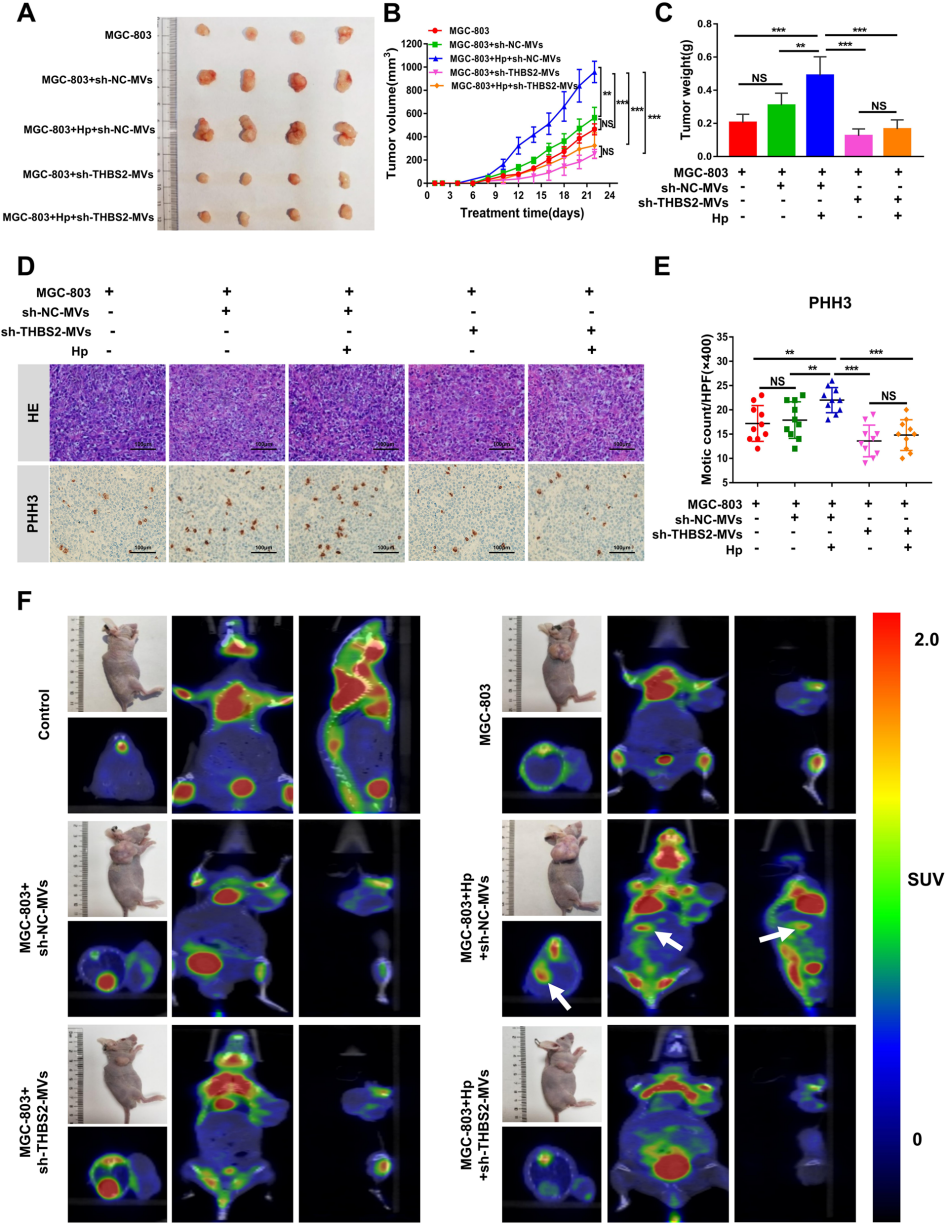
Depletion of the THBS2 gene reduces the tumor-promoting ability of BMSC-MVs in nude mouse xenografts. **A**-**C** Representative images of tumor, tumor size and tumor weight in nude mice 3 weeks after subcutaneous injection of MGC-803 cells alone, MGC-803 cells cocultured with sh-NC-MVs, MGC-803 cells cocultured with Hp + sh-NC-MVs, MGC-803 cells cocultured with sh-THBS2-MVs, and MGC-803 cells cocultured with Hp + sh-THBS2-MVs. **D**, **E** Representative PHH3 IHC images and quantitative analysis of the PHH3 index of tumor sections from different groups of nude mice are shown. **F** Representative PET-CT images of nude mice 3 months after subcutaneous injection with MGC-803 cells alone, MGC-803 cells cocultured with sh-NC-MVs, MGC-803 cells cocultured with Hp + sh-NC-MVs, MGC-803 cells cocultured with sh-THBS2-MVs, and MGC-803 cells cocultured with Hp + sh-THBS2-MVs. The white arrow indicates suspected abdominal metastasis of xenograft tumors. BMSCs: bone marrow-derived mesenchymal stem cells; MVs: microvesicles; Hp: *Helicobacter pylori;* GC: gastric cancer; IHC: immunohistochemistry. **P < .01, ***P < .001, ****P < .0001, NS: no significance

The role of sh-THBS2-BMSC-MVs in the tumorigenesis of *H. pylori*-associated GC was evaluated using a subcutaneous xenograft tumor model in nude mice. Three weeks after injection, the weight and volume of the subcutaneous tumor in the Hp + sh-THBS2-BMSC-MVs + MGC-803 group were significantly decreased compared with those in the Hp + sh-NC-BMSC-MVs + MGC-803 group (*P* < 0.001, Fig. 6A-C). In addition, the results of immunohistochemical staining revealed that the number of mitotic cells was decreased in the Hp + sh-THBS2-BMSC-MVs + MGC-803 group compared with the Hp + sh-NC-BMSC-MVs + MGC-803 group (*P* < 0.001, Fig. 6D, E).

**Fig. 7:**
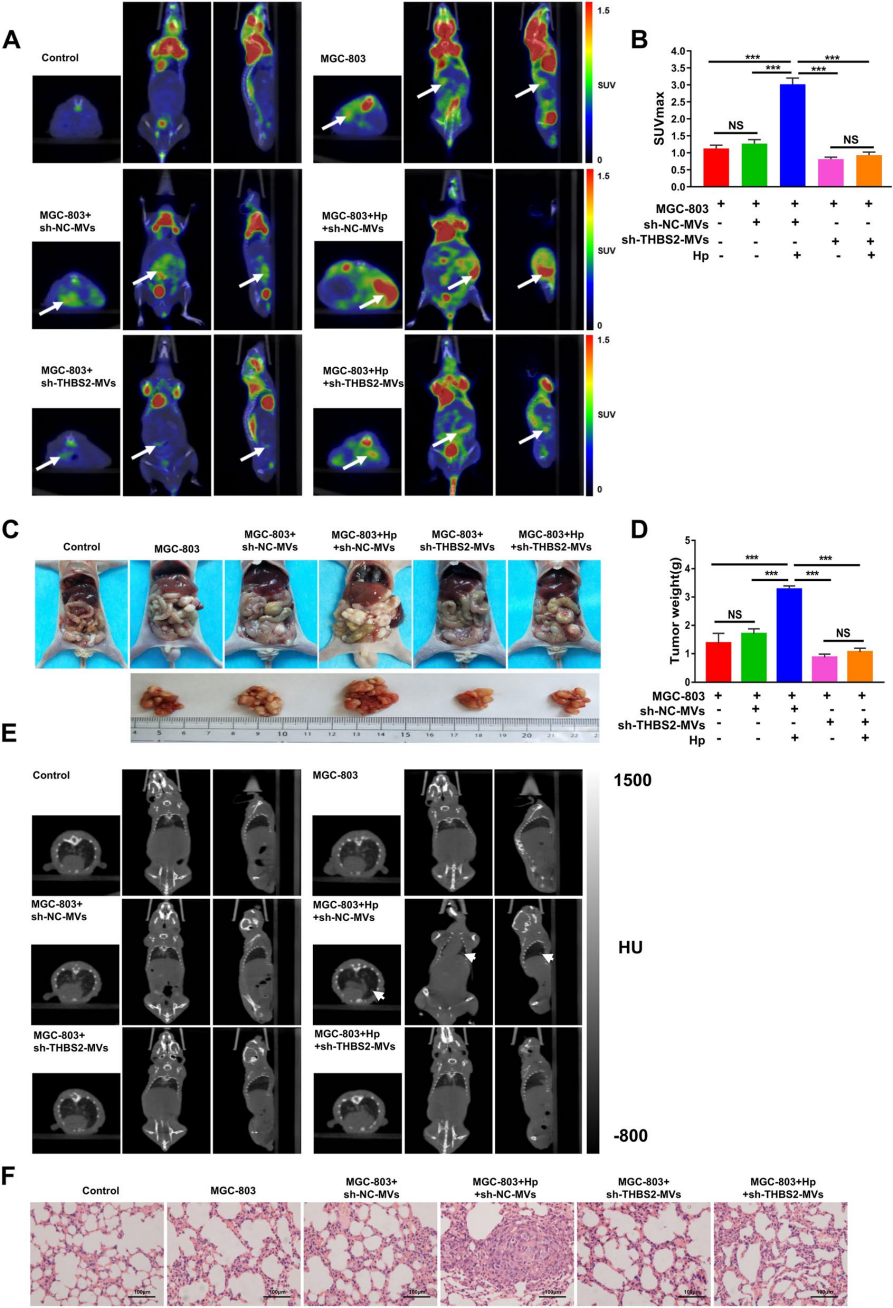
Depletion of the THBS2 gene reduces the tumor-promoting ability of BMSC-MVs in nude mouse xenografts. **A **Representative PET-CT images of nude mice 4 weeks after intraperitoneal injection with MGC-803 cells alone, MGC-803 cells cocultured with sh-NC-MVs, MGC-803 cells cocultured with Hp + sh-NC-MVs, MGC-803 cells cocultured with sh-THBS2-MVs, and MGC-803 cells cocultured with Hp + sh-THBS2-MVs. The white arrow indicates suspected abdominal metastasis of xenograft tumors. **B** The SUV_max_ values of intraperitoneal metastases in different groups of nude mice. **C**, **D** Representative images and tumor weights of intraperitoneal metastatic tumors in different groups of nude mice. **E** Representative CT images of nude mice 4 weeks after tail vein injection with MGC-803 cells alone, MGC-803 cells cocultured with sh-NC-MVs, MGC-803 cells cocultured with Hp + sh-NC-MVs, MGC-803 cells cocultured with sh-THBS2-MVs, and MGC-803 cells cocultured with Hp + sh-THBS2-MVs. **F** H&E pathological images of lung tissue 4 weeks after tail vein injection in different groups of nude mice. BMSCs: bone marrow-derived mesenchymal stem cells; MVs: microvesicles; Hp: *Helicobacter pylori;* GC: gastric cancer; ***P < .001, NS: no significance

To evaluate the effect of sh-THBS2-BMSC-MVs on the metastasis of GC xenografts in nude mice, we generated a nude mouse abdominal cavity metastasis model, a subcutaneous transplanted tumor distant metastasis model, and a tail vein injection metastasis model. Three months later, 18F-FDG PET/CT was performed on the nude mice with subcutaneously transplanted tumors. Abdominal metastasis was detected in the Hp + sh-NC-MVs + MGC-803 group, while no obvious distant metastasis was observed in the Hp + sh-THBS2-MVs + MGC-803 groups (Fig. 6F). Four weeks after transplantation, 18F-FDG PET/CT was applied to the nude mice with abdominal transplanted tumors. Abdominal metastasis was detected in the transplantation group, and glucose metabolism in the Hp + sh-THBS2-MVs + MGC-803 group was significantly lower than that in the Hp + sh-NC-MVs + MGC-803 group (*P* < 0.001, Fig. 7A, B). In addition, the size and weight of abdominal metastases were decreased in the Hp + sh-THBS2-MVs + MGC-803 group (*P* < 0.001, Fig. 7C, D). Four weeks later, CT was used to scan the vein injection metastasis model mice. The results showed suspicious lung tumors only in the Hp + BMSC-MVs + MGC-803 group (Fig. 7E, F). Taken together, these results suggested that depletion of the THBS2 gene can reduce the tumor-promoting ability of BMSC-MVs in nude mouse xenografts.

## Discussion

In recent years, accumulating evidence has demonstrated that BMSCs play an important role in the initiation, progression and metastasis of certain tumor types [5, 28–31], and paracrine factors secreted by BMSCs may be involved in these responses [31–33]. Extracellular vesicles, as paracrine factors, carry substantial populations of bioactive molecules, transfer their contents to neighboring tumor cells, and induce phenotypic modifications in recipient cells [30, 34]. As the EV research field continues to mature, evidence for the important roles of MVs in microenvironment modulation has become compelling. In addition to soluble factors, BMSC-MVs have also been proposed as a new mechanism underlying the paracrine action of BMSCs. Although research on the interaction between BMSC-derived exosomes and tumor growth has gained attention in recent years, the effects of BMSC-MVs on the initiation and progression of tumors remain relatively unexplored, and the effects of BMSC-MVs on GC remain unclear.

The present study demonstrated that BMSCs can significantly enhance MV production by *H. pylori* stimulation. These results indicate that *H. pylori* may regulate the release of MVs. Furthermore, in the present study, the role of MVs from BMSCs treated with *H. pylori* in the progression of gastric cancer was initially observed. MVs from BMSCs treated with *H. pylori* can promote the proliferation and migration of MGC-803 gastric cancer cells in vitro and accelerate tumor growth in vivo. Furthermore, these present results indicate that THBS2 is enriched in MVs from BMSCs treated with *H. pylori* and can be delivered to MGC-803 gastric cancer cells, reprogramming MGC-803 GC cells in the tumor microenvironment to promote the growth and metastasis of GC.

EVs, specifically exosomes and MVs, are presumed to play key roles in cell–cell communication via the transfer of biomolecules between cells. The biogenesis of these two types of EVs differs, as they originate from either endosomal (exosomes) or plasma (MVs) membranes. Both EV types encapsulate reporter proteins and mRNA, but only MVs transfer the reporter function to recipient cells. Kanada et al. [35] reported that exosomes and MVs are structurally and functionally distinct. MVs can target recipient cells and affect the functions of recipient cells by delivering RNAs, lipids and proteins. Recent studies have indicated that shedding vesicles contribute to key biological processes, including membrane trafficking and the horizontal transfer of cargos (RNA and proteins [36]) to recipient target cells [37]. A number of biological molecules can be transferred by MVs to recipient cells, leading to the exchange of genetic information and the reprogramming of recipient cells. MVs may change functional target cells by delivering intracellular proteins. For example, MVs released from endothelial cells can promote angiogenesis through the transfer of proangiogenic molecules, such as growth factors and their activators [38]. These vesicles may also horizontally transfer genetic information to target cells [39]. The mechanism involved may be that MVs directly transfer THBS2 genetic material to MGC-803 gastric cancer cells.

THBS is a multifunctional glycoprotein released from various types of cells, including stromal fibroblasts, endothelial cells, and immune cells [21, 40]. THBS2 exerts its diverse biological effects, such as effects on angiogenesis, cell motility, apoptosis and cytoskeletal organization, by binding with ECM proteins and cell surface receptors [22, 41, 42]. Wang et al. [43] suggested that THBS2 may be a prognostic biomarker of colorectal cancer. In colorectal cancer, THSB2 mRNA levels were reported to be elevated compared to those of normal tissues: the analysis of the published gene expression data from the Cancer Genome Atlas indicated that the expression increases with the tumor stage and node involvement. Cao et al. [24] analyzed samples obtained from 20 GC patients who underwent tumor resection at the Department of Abdominal Surgery and found that the THBS2 gene was significantly upregulated in tumor tissues (***P < 0.001). THBS2 was identified as a GC biomarker. Zhuo et al. [23] identified 43 genes with altered expression in gastric cancer by analyzing 105 pairs of tumor tissues and the corresponding adjacent normal tissues. These researchers observed that THBS2 was overexpressed in gastric tumor tissues and proposed THBS2 as a prognostic indicator of gastric cancer, in which high THBS2 expression was correlated with poor prognosis.

In the present study, we found that THBS2 was highly expressed in Hp + BMSC-MVs. We also discovered that Hp + BMSC-MVs can efficiently incorporate THBS2 into MGC-803 GC cells, which enhanced the proliferation, migration, invasion and metastasis of MGC-803 GC cells, while the knockdown of THBS2 in BMSCs reversed this phenomenon. This finding suggests that Hp + BMSC-MVs can deliver numerous active THBS2 molecules to gastric cancer cells, thereby affecting the function of GC cells and consequently promoting the growth and metastasis of GC.

## Conclusion

Overall, these present findings demonstrate that MVs from BMSCs treated with *H. pylori* can promote the proliferation, migration, invasion and metastasis of gastric cancer cells. MVs secreted by BMSCs treated with *H. pylori* can transport THBS2 to GC cells and reprogram cells in the tumor microenvironment to promote the growth and metastasis of GC. Therefore, THBS2 may be a potential therapeutic target for H pylori-associated GC.

### Supplementary Information

**Additional file 1:** **Supporting Figure 1.** Phenotype identification and induced differentiation of BMSCs. A: Flow cytometry identifies the phenotype of BMSCs. CD44, CD73, CD105, and Sca-1 are positive, and CD11b and CD45 are negative. B: BMSCs are induced to differentiate into osteogenic, fat, and cartilage cells.

**Additional file 2:** **Supporting Figure 2.** Characterization of microvesicles. A-C: Diameter and concentration of microvesicles analyzed by NTA. D: Representative immunoblot bands of CD9, CD81 and TSG101 in BMSC-sh-NC-MVs, Hp+BMSC-sh-NC-MVs, BMSC-sh-THBS2-MVs, and Hp+BMSC-sh-THBS2-MVs. BMSCs: bone marrow-derived mesenchymal stem cells; MVs: microvesicles; Hp: Helicobacter pylori; NTA: nanoparticle tracking analysis.

**Additional file 3.** 

**Additional file 4.** 

## Data Availability

The raw data generated and analyzed in the current study are not publicly available but are available from the corresponding author on reasonable request.

## Abbreviations

BMSCs: Bone marrow-derived mesenchymal stem cells
EVs: Extracellular vesicles
MVs: Microvesicles
ECM: Extracellular matrix
*H pylori*: *Helicobacter pylori*
THBS2: Thrombospondin-2
GC: Gastric cancer
CAFs: Cancer-associated fibroblasts
NTA: Nanoparticle Tracking Analysis
FCM: Flow cytometry
