# Supplementary material for: Microvesicles from bone marrow-derived mesenchymal stem cells promote Helicobacter pylori-associated gastric cancer progression by transferring thrombospondin-2
Source: Cell Commun Signal. 2023 Oct 5;21:274. doi: 10.1186/s12964-023-01127-y (PMC10552243; doi:10.1186/s12964-023-01127-y)
Supplement: Supplementary file 5 — Additional file 4. [file 12964_2023_1127_MOESM4_ESM.docx]

**Supplementary methods**

**Cell lines and cell culture**

The BMSCs was collected from four-week-old BALB/c mice (Beijing Huafukang Biotechnology Co., Ltd, Beijing, China). Cells were cultured and expanded in low glucose Dulbecco’s modified Eagle’s medium (DMEM, Gibco) with 10% fetal bovine serum (FBS, Gibco) at 37°C in humidified incubator with 5% CO_2_. BMSCs at passage six were used for the detection of immunophenotype and multipotent differentiation potentials. The human gastric adenocarcinoma cell line MGC-803 was purchased from Shanghai Zhongqiao Xinzhou Biological Technology Co., Ltd. (Shanghai, China). These cells were cultured in RPMI-1640 with 10% fetal bovine serum (FBS, Gibco) at 37°C in a humidified cell incubator.

**The assay for cell proliferation**

MGC-803 cells were seeded into a 96-well at a concentration of 1×10^3^ cells/well. These cells were treated with MVs at different concentrations for 12 hours. Then CCK-8 was added and incubated for one hour. The OD value at 450 nm was detected using Enzyme-labelled meter. ​In order to track the rate of cell proliferation, cells were labeled with 5-(and-6)-carboxyfluorescein diacetate succinimidyl ester (CFSE, Ebioscience) at room temperature for 10 minutes in the dark. Then, the reaction was quenched with cold complete media. CFSE-labeled cells were transferred to a 12-well at a concentration of 1×10^5^ cells/well. Afterwards, these cells were treated with MVs (0.25 μg/ml) for 12 hours. Finally, cells were collected and subjected to flow cytometry analysis at 488nm.

**Transwell migration and invasion assays**

The MGC-803 (1×10^5^ cells) with MVs were seeded onto the upper compartment of the Transwell chamber (8.0 μm, 24-well, Corning, NY, USA). After 12 hours, the cells at the upper chamber were removed using a cotton swab. Then, cells that migrated through the pores were fixed with 4% paraformaldehyde, and stained with crystal violet. For the invasion assay, the upper compartment was covered with 40 µl matrigelm and other protocols were similar to the migration assay. Then, these cells were observed under a microscope, and at least six fields of cells were assessed for each group. Each assay was repeated for three times.

**Protein preparation and western blot analysis**

The samples were harvested using radioimmunoprecipitation assay (RIPA) lysis buffer, and quantified using the bicinchoninic acid (BCA) protein assay kit (Vazyme, China). Then, the protein extracts were separated by sodium dodecyl sulfate-polyacrylamide gel electrophoresis (SDS-PAGE), and transferred onto polyvinylidene fluoride (PVDF) membranes. The blocking of the membranes was performed using 10% non-fat milk for 60 minutes. Then, these were incubated at 4°C overnight with antibodies against the following proteins: anti-CD9 (1:1000, Abcam), anti-CD8l (1:1000, Gene Tex), anti-TSG101 (1:1000, Proteintech), anti-Thrombospondin-2 (1:500, Abcam), anti-β-actin (1:2000, Abcam), and anti-GAPDH (1:2000, Abcam). Following the incubation with the secondary antibodies, the signals were visualized using the enhanced chemiluminescence imaging system (UVP, USA).

**Immunofluorescence**

A red fluorescent dye (DiI; Beyotime) was applied to label MVs, in accordance to the instructions of the manufacturer. Centrifuge at 20,000 g for 75 minutes at 4°C to remove the excess dye. A green fluorescent dye (DiO; Beyotime) was used to label cell membrane. Then the DiO-labeled MGC-803 cells were incubated with DiI-labeled MVs for observation. The cell nuclei were stained with 4′,6-diamidine-2′-phenylindole dihydrochloride (DAPI) and the specimens were observed by a fluorescent microscope (Olympus, Tokyo, Japan).

**Histology and immunohistochemistry**

Hematoxylin and eosin (H&E) staining and immunohistochemistry were performed on sections obtained from the paraffin-embedded tumors. These tissue sections were de-waxed in xylene, and rehydrated in graded alcohol. Then, the endogenous peroxidase activity was eliminated by incubating with 0.3% hydrogen peroxide (H_2_O_2_). Antigen-retrieval was performed by incubating in citrate buffer (pH 6.0). Non-specific binding was blocked by 5% bovine serum albumin, and the tissue sections were incubated with specific antibodies. Finally, the slices were mounted with neutral gum for microscopic examination, and cells with brown granules in the cytoplasm or nucleolus were considered positive.
